# Innovative CQD detector for broadband multispectral imaging

**DOI:** 10.1038/s41377-024-01621-z

**Published:** 2024-11-12

**Authors:** Shengli Sun, Yaran Li, Fansheng Chen

**Affiliations:** 1 National Key Laboratory of Infrared Detection Technologies, Shanghai Institute of Technical Physics, Chinese Academy of Sciences, Shanghai, China; 2 State Key Laboratory of Infrared Physics, Shanghai Institute of Technical Physics, Chinese Academy of Sciences, Shanghai, China; 3 Key Laboratory of Intelligent Infrared Perception, Chinese Academy of Sciences, Shanghai, China

**Keywords:** Optoelectronic devices and components, Mid-infrared photonics, Imaging and sensing

*Nature Photonics* **18**, 1147–1154 (2024)

10.1038/s41566-024-01492-1

Multispectral imaging, encompassing a broad spectrum ranging from the visible to the infrared band, plays a pivotal role in various applications, including target identification, compositional analysis, biomedical diagnosis, environmental remote sensing, and many others. Traditional mid- and short-wave infrared detectors, such as InGaAs, InSb, and HgCdTe detectors, encounter challenges in achieving wide-spectrum coverage, primarily due to the physical limitations of the detector materials and the complexities inherent in their structures. Professor Hao’s team from Beijing Institute of Technology has introduced an innovative vertically stacked PbS/HgTe colloidal quantum dot (CQD) detector featuring optimized graded energy gaps. This unique configuration and its underlying physical mechanisms facilitate precise control of photogenerated carriers within the multilayered stacked structure, ultimately resulting in a broadband response spanning from 0.5 to 4.0 μm. By harnessing this technique, a high-performance CQD focal plane array with dimensions of 640 × 512 and a pixel pitch of 15 μm has been fabricated and characterized, demonstrating exceptional sensitivity, good uniformity, and robust temperature adaptability. This technique holds immense significance for further expanding the response spectrum of detectors, enabling multispectral imaging, and facilitating the integration and miniaturization of photoelectric systems. In the realm of infrared detector production and manufacturing, this technique offers a clear pathway for reducing manufacturing costs and achieving stable, scaled-up mass production.

